# Androgen deprivation therapy for prostate cancer and the risk of hematologic disorders

**DOI:** 10.1371/journal.pone.0229263

**Published:** 2020-02-19

**Authors:** Jui-Ming Liu, Yueh-Ping Liu, Heng-Chang Chuang, Chun-Te Wu, Yu-Li Su, Ren-Jun Hsu

**Affiliations:** 1 Division of Urology, Department of Surgery, Taoyuan General Hospital, Ministry of Health and Welfare, Taoyuan, Taiwan; 2 Department of Medicine, National Yang-Ming University, Taipei, Taiwan; 3 Graduate Institute of Life Sciences, National Defense Medical Center, Taipei, Taiwan; 4 Department of Emergency Medicine, National Taiwan University Hospital, Taipei, Taiwan; 5 Department of Medical Affairs, Ministry of Health and Welfare, Taipei, Taiwan; 6 Department of Urology, Chang Gung Memorial Hospital, Keelung, Taiwan; 7 Division of Hematology-Oncology, Department of Internal Medicine, Chang Gung Memorial Hospital, Kaohsiung, Taiwan; 8 Clinical Trial Center, Chang Gung Memorial Hospital, Kaohsiung, Taiwan; 9 Cancer Research Center, Hualien Tzu Chi Hospital, Buddhist Tzu Chi Medical Foundation, Hualien, Taiwan; 10 College of Medicine, Tzu Chi University, Hualien, Taiwan; Medizinische Universitat Innsbruck, AUSTRIA

## Abstract

**Purpose:**

This study aimed to investigate the association between androgen deprivation therapy (ADT) and the risk of subsequently developing hematologic disorders in patients with prostate cancer.

**Materials and methods:**

This population-based nationwide cohort study utilized data from the Taiwan National Health Insurance Research Database between 1997 and 2013. The patients were divided into three groups—those who received ADT only (ADT-only group), those who had radiotherapy (RT) only (RT-only group), and those treated with radical prostatectomy (RP) only (RP-only group). The study outcome was newly diagnosed hematologic disorder, including anemia and hematologic malignancy. Propensity score-matched, Cox regression, and Kaplan–Meier curve analyses were performed to investigate the risk of subsequently developing hematologic disorders after ADT.

**Results:**

Of the 17,168 patients with prostate cancer who were included in the study, 13,318 met the inclusion and exclusion criteria. After propensity score matching, 1,797, 1,797, and 1,797 patients treated with ADT only, RT only, and RP only, respectively, who had a median follow-up period of 4.32 years were included in the study cohort. Compared with the patients treated with RP only, those who received ADT and RT were significantly at increased risk of subsequently developing hematologic disorders (ADT: adjusted hazard ratio [aHR]: 1.60, 95% confidence interval [CI]: 1.29–1.97; RT: aHR, 1.98, 95% CI: 1.62–2.42) according to the Cox regression analysis. Based on the Kaplan–Meier curve analysis, patients with bone metastasis who received ADT only had the lowest cumulative probabilities of not developing hematologic disorders. Moreover, a significantly increased risk of hematologic disorders was observed with the increasing duration of ADT (*P* for trend < .001).

**Conclusions:**

The use of ADT in patients with prostate cancer may increase the risk of subsequently developing hematologic disorders.

## Introduction

Prostate cancer (PCa) is the second most common cancer among men worldwide, with an annual incidence of approximately more than 1.1 million. [1] Androgen deprivation therapy (ADT) has been a mainstay treatment for advanced PCa for more than 70 years. [2] However, it is associated with several adverse effects, such as osteoporosis, metabolic syndrome, and cardiovascular diseases. [3,4]

By stimulating the release of erythropoietin and increasing bone marrow activity and iron incorporation, androgen may stimulate erythropoiesis. [5,6] Moreover, it has been used for bone marrow failure treatment. [7] Meanwhile, an association between ADT and anemia has been reported. [8,9]

Non-Hodgkin’s lymphoma (NHL) is one of the most common hematologic malignancies worldwide, with an incidence significantly higher in men than in women.[10] Moreover, the increased risk of hematologic malignancy after radiotherapy for PCa has been found. [11,12] Nonetheless, the relationship between androgen and ADT as well as hematologic malignancy has not been completely understood. Moreover, hematologic disorders may reduce quality of life and can be a poor prognostic factor in patients with PCa.[13]

However, studies about the association between ADT and hematologic disorders are limited to date. Thus, this study used data from a large-scale nationwide database of Taiwan to determine whether the use of ADT is associated with the subsequent development of hematologic disorders, including anemia and hematologic malignancy, in patients with PCa.

## Materials and methods

### Data source and collection

This nationwide cohort study utilized data from the National Health Insurance Research Database (NHIRD) of Taiwan. Moreover, a sub-database of the NHIRD referred to as the Registry for Catastrophic Illness Patient Database (RCIPD) was used. Patients with major diseases, including malignant neoplasms, received waivers for medical payments after receiving certification for having a catastrophic illness, with the relevant diagnoses confirmed by experts assigned by the NHI administration by reviewing clinical information, pathological reports, and imaging findings. All medical diagnoses included in the NHIRD and RCIPD were determined according to the International Classification of Diseases, 9th revision, Clinical Modification (ICD-9-CM) codes. The Institutional Review Board of the Tri-Service General Hospital had approved this study (approval number: TSGHIRB NO B-104-21). As this was a retrospective study and all data were anonymized, the Institutional Review Board waived the need to obtain patient consent.

### Study population

**Fig 1:**
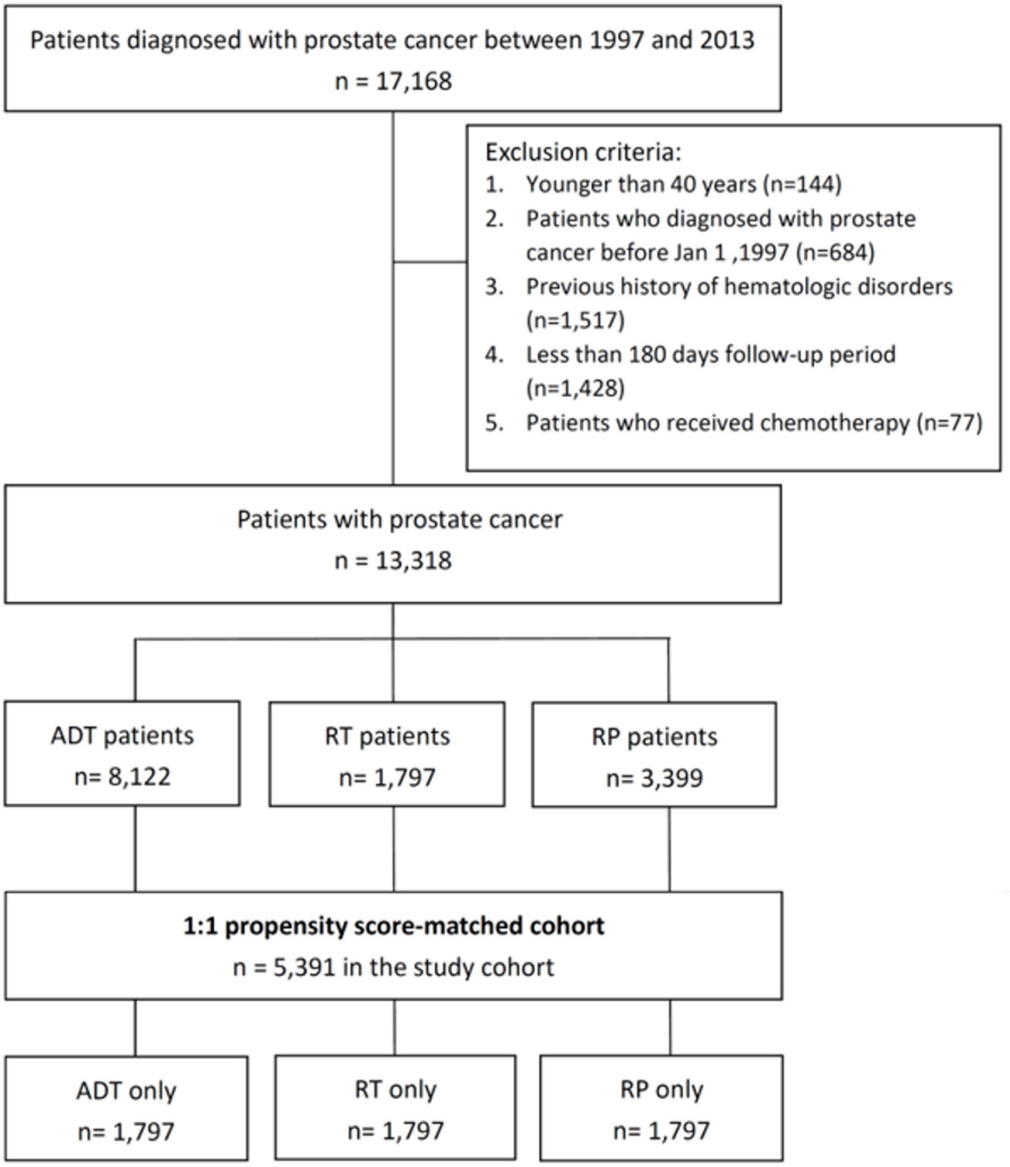
Study flowchart of cohort selection.

The participants were selected by utilizing the RCIPD data from January 1997 to December 2013. The accuracy of PCa diagnoses was confirmed using both the ICD-9-CM codes and the inclusion criteria in the RCIPD. In this study, we only enrolled patients with PCa who had a follow-up period of more than 180 days after the initial diagnosis of PCa. Moreover, those who were newly diagnosed with PCa (ICD-9-CM: 185) [14,15] between January 1997 and June 2013 were included. The exclusion criteria were as follows: patients diagnosed with PCa before January 1, 1997, those younger than 40 years at the time of diagnosis, those who had a previous history of hematologic disorders, and those who had less than 180 days of follow-up after the diagnosis of PCa. Moreover, patients who received chemotherapy were not included (Fig 1).

The participants were divided into three groups according to the different therapeutic approaches used for treatment: ADT-only group, patients who received ADT only; RT-only group, those who received radiotherapy (RT) only; and RP-only group, those treated with radical prostatectomy (RP) only. Patients who only underwent ADT were classified as the ADT-only group. Patients who only received radiotherapy without any other therapeutic approaches for PCa were classified as the RT-only group, and those who only received radical prostatectomy were classified as the RP-only group.

### Study outcomes and covariates

PCa patients who received ADT were clearly identified in the RCIPD. The different types of ADT used were based on the utilization of gonadotropin-releasing hormone (GnRH) agonists (leuprolide, goserelin, buserelin, and triptorelin), oral antiandrogens (cyproterone acetate, bicalutimide, flutamide, and nilutamide), and estrogens (diethylstilbestrol and estramustine).

The study outcome wass were newly diagnosed hematologic disorders, including anemia and hematologic malignancy, which have been associated with poor prognosis in patients with PCa. [12] The new diagnoses of hematologic malignancies listed in the RCPID were identified using the relevant ICD-9-CM codes (S1 Table). The diagnoses of anemia were identified using both the relevant ICD-9-CM codes and the receipt of further treatments (including iron supplements, vitamin B12, folic acid, erythropoietin, or blood transfusion). Patients treated with ADT were further categorized into two groups according to the presence of bone metastasis (ADT patients with bone metastasis and ADT patients without metastasis) to evaluate the impact of bone metastasis on the development of hematologic disorders. [12]

The actual occurrence of hematologic disorders in patients treated with ADT was identified after the initiation of ADT and after more than 180 days since the PCa diagnosis. Meanwhile, the occurrence of hematologic disorders in the RP-only and RT-only groups was confirmed only after more than 180 days since the PCa diagnosis and after the patients had undergone RP or completed RT. Censoring was defined as the date of incidence of hematologic disorders, death, loss to follow-up, or until the end of the follow-up period (December 31, 2013), whichever came first.

We calculated the mean age ± standard deviation and divided the patients into five groups according to age. The covariates assessed included alcohol abuse, tobacco use disorder, obesity, diabetes mellitus, hypertension, hyperlipidemia, coronary heart disease, chronic kidney disease, cerebral vascular accident, chronic liver disease, comorbidities related to increased risk of anemia (Crohn’s disease, rheumatoid arthritis, ulcerative colitis, previous history of gastrointestinal bleeding) (S1 Table), and use of medications associated with increased risks of bleeding (antiplatelets, anticoagulants, and nonsteroidal anti-inflammatory drugs).

### Statistical analysis

The demographic characteristics of the three groups were presented as total number (n) and percentage (%) for categorical variables as well as mean and standard deviation (SD) for continuous variables. We used the analysis of variance and chi-square test to compare continuous and categorical variables between the groups. The primary endpoint was new diagnosis of anemia or hematologic malignancy. Patient mortality was considered a competing risk. The survival curves were presented as Kaplan–Meier (KM) curves, which were assessed using log-rank test. Risk factors, including demographic data, comorbidities, and use of medications for anemia or hematologic malignancy, were assessed using a Cox proportional hazards model with competing risks, and hazards ratios (HRs) with 95% confidence intervals (CIs) were evaluated. Moreover, we calculated the incidence rates (IRs, per 1,000 person-year) of anemia or hematologic malignancy. A 1:1 propensity score matching was performed to eliminate potential confounders, and secondary analysis was conducted on patients who did not receive ADT, which was represented by the RP-only group. We assessed whether the risk of developing hematologic disorders varied according to the different types of ADT used.

### Sensitivity analyses

We used several sensitivity tests in this study. First, patients who received chemotherapy, which has a significant impact on hematopoiesis, were excluded. Second, we exclusively defined hematologic malignancies as hematologic malignancies diagnosed in patients who were registered in the RCIPD, which included those with a catastrophic illness confirmed via a detailed examination conducted by experts. Furthermore, to ensure that the diagnoses were accurate, anemia was strictly defined as patients who both had a relevant ICD-9-CM code and received further treatments. Third, we performed a 1:1 nearest-neighbor propensity score-matched analysis to minimize the influence of covariates. Fourth, competing risk regression models were utilized to adjust for the death of any participants. [16] Fifth, we further divided the use of ADT into three groups, namely, no ADT use (represented by the RP group),<12 months of ADT use, and ≥ 12 months of ADT use, to investigate the effect of the duration of ADT on the development of hematologic disorders. Finally, falsification analysis was conducted to validate the associations between the use of ADT and other unmeasured comorbidities. [17]

All statistical analyses were performed using SAS version 9.2 (SAS Institute, Cary, NC, USA) and STATA version 11.0 (StataCorp, College Station, TX, USA). A two-tailed *p* value < 0.05 was considered significant.

## Results

In total, 13,318 patients who were newly diagnosed with PCa and who met all the inclusion criteria were enrolled in this study from January 1997 to June 2013. Among the participants, 8,122, 1,797, and 3,399 were treated with ADT only, RT only, and RP only, respectively. After the propensity score matching, there were 1,797, 1,797, and 1,797 patients who were treated with ADT only, RT only, and RP only, respectively. In total, 787 patients developed hematologic disorders during a median follow-up period of 4.32 years (interquartile range: 2.11–7.74) years.

The demographic characteristics of the participants are shown in Table 1. The IRs of hematologic disorders in the ADT, RT, and RP groups were 28.18, 36.01, and 17.07 per 1000 person-years, respectively (Table 2). Meanwhile, the IRs of hematologic malignancy in the ADT, RT, and RP groups were 1.93, 3.96, and 1.80 per 1000 person-years, respectively.

**Table 1:** Demographic characteristics of prostate cancer patients according to use of androgen deprivation therapy, radiotherapy, and radical prostatectomy.

	Full cohort				Propensity Score–Matched Cohort			
Variables No. (%)	ADT	RT	RP	*P*-value	ADT	RT	RP	*P*-value
**Total**	8122	1797	3399		1797	1797	1797	
**Age (mean±SD, years)**	71.28±9.66	72.54±7.92	67.50±10.63	<0.001**	72.63±8.53	72.54±7.92	72.64±8.26	0.923
**Age at diagnosis**				<0.001**				0.126
<50	181 (2.23)	19 (1.06)	165 (4.85)		20 (1.11)	19 (1.06)	14 (0.78)	
50–60	911 (11.22)	109 (6.07)	682 (20.06)		124 (6.90)	109 (6.07)	112 (6.23)	
60–70	2266 (27.90)	483 (26.88)	1147 (33.76)		489 (27.21)	483 (26.88)	508 (28.27)	
70–80	3251 (40.02)	893 (49.69)	961 (28.27)		817 (45.47)	893 (49.69)	822 (45.74)	
>80	1513 (18.63)	293 (16.30)	444 (13.06)		347 (19.31)	293 (16.30)	341 (18.98)	
**Follow-up period** **(mean±SD, years)**	6.01 (4.34)	5.16 (3.70)	5.31 (3.93)	<0.001**	5.89 (4.29)	5.16 (3.70)	5.05 (3.83)	<0.001**
**Comorbidity**								
Alcohol abuse	16 (0.20)	4 (0.22)	4 (0.12)	0.592	10 (0.56)	4 (0.22)	2 (0.11)	0.038*
Tobacco use disorder	1108 (13.64)	266 (14.80)	362 (10.65)	<0.001**	228 (12.69)	266 (14.80)	240 (13.36)	0.167
Obesity	15 (0.18)	7 (0.39)	7 (0.21)	0.238	6 (0.33)	7 (0.39)	4 (0.22)	0.661
Diabetes mellitus	1586 (19.53)	333 (18.53)	617 (18.15)	0.193	319 (17.75)	333 (18.53)	317 (17.64)	0.750
Hypertension	3964 (48.81)	938 (52.20)	1449 (42.63)	<0.001**	936 (52.09)	938 (52.20)	928 (51.64)	0.939
Hyperlipidemia	1518 (18.69)	367 (20.42)	707 (20.80)	0.018*	336 (18.70)	367 (20.42)	325 (18.09)	0.180
Coronary heart disease	1706 (21.00)	412 (22.93)	553 (16.27)	<0.001**	363 (20.20)	412 (22.93)	363 (20.20)	0.068
Chronic kidney disease	390 (4.80)	101 (5.62)	165 (4.85)	0.340	72 (4.01)	101 (5.62)	103 (5.73)	0.031*
Chronic liver disease	891 (10.97)	188 (10.46)	344 (10.12)	0.382	158 (8.79)	188 (10.46)	173 (9.63)	0.237
Cerebral vascular accident	1086 (13.37)	217 (12.08)	3553(10.39)	<0.001**	199 (11.07)	217 (12.08)	228 (12.69)	0.321
Crohn’s disease	65 (0.80)	16 (0.89)	25 (0.74)	0.834	17 (0.95)	16 (0.89)	9 (0.50)	0.254
Ulcerative colitis	5 (0.06)	0 (0.00)	1 (0.03)	0.475	2 (0.11)	0 (0.00)	0 (0.00)	0.135
Rheumatoid arthritis	98 (1.21)	23 (1.28)	21 (0.62)	0.012*	26 (1.45)	23 (1.28)	15 (0.83)	0.215
GI bleeding	359 (4.42)	92 (5.12)	122 (3.59)	0.024*	72 (4.01)	92 (5.12)	68 (3.78)	0.107
**Medication use**								
Antiplatelet	5550(68.33)	1161(64.61)	1607(47.28)	<0.001**	1247(69.39)	1161(64.61)	980(54.54)	<0.001**
Anticoagulation	539 (6.64)	126 (7.01)	137 (4.03)	<0.001**	108 (6.01)	126 (7.01)	92 (5.12)	0.058
NSAID	4136(50.92)	740(41.18)	183 (5.38)	<0.001**	887(49.36)	740(41.18)	100 (5.56)	<0.001**

Abbreviations: ADT, androgen deprivation therapy; RT, radiotherapy; RP, radical prostatectomy; GI, gastrointestinal; SD, standard deviation.

*P <0.05

**P<0.001

**Table 2:** Incidence (per 1000 person-years) of hematologic disorders (anemia or hematologic malignancy) among ADT, RT, and RP groups.

	Incidence (per 1000 person-years)			ADT v.s. RP		RT v.s. RP	
Outcome	ADT (n = 1,797)	RT (n = 1797)	RP (n = 1797)	Adjusted HR (95% CI)	*P*-value	Adjusted HR (95% CI)	*P*-value
Hematologic disorders	28.18	36.01	17.07	1.60 (1.29–1.97)	<0.001**	1.98 (1.62–2.42)	<0.001**
Anemia	27.20	33.08	16.05	1.63 (1.31–2.02)	<0.001**	1.92 (1.56–2.37)	<0.001**
Hematologic malignancy	1.93	3.96	1.80	1.12 (0.53–2.37)	0.760	2.48 (1.29–4.76)	0.006*

Abbreviations: ADT, androgen deprivation therapy; RT, radiotherapy; RP, radical prostatectomy; HR, hazard ratio; CI, confidence interval

*P <0.05

**P <0.001

We performed the falsification analysis to validate the lack of statistically significant associations (S2 Table).

A significantly increased risk of subsequently developing hematologic disorders was observed in the ADT group compared with the RP group after adjusting for age, comorbidities, and use of medications (aHR = 1.60; 95% CI = 1.29–1.97; *P* < .001) (Table 2). In addition, the RT-only group had a higher risk of developing hematologic disorders. Age, chronic kidney disease, and use of antiplatelets were the significant risk factors for hematologic disorders, as shown in S3 Table. The results of the Cox regression analysis of the risk of hematologic disorders in patients treated with ADT who presented with bone metastasis and those without, patients who received RT, and patients treated with RP are shown in S4 Table. The patients treated with ADT who presented with bone metastasis were significantly at increased risk of subsequently developing hematologic disorders (aHR = 2.87; 95% CI = 2.08–2.97; *P* < .001) compared with those who underwent RP.

**Fig 2:**
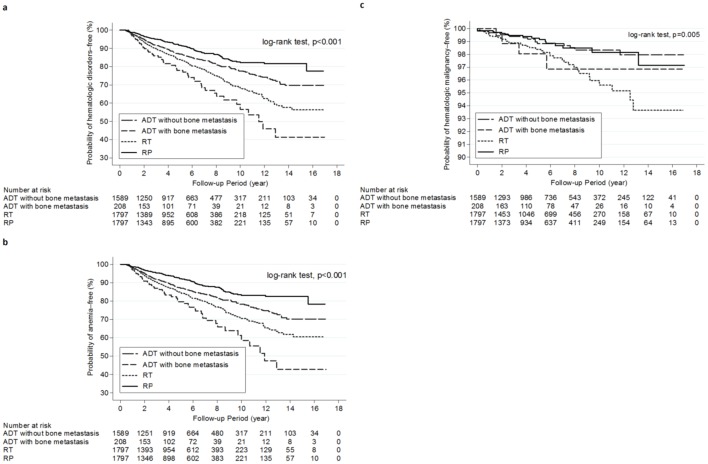
Kaplan-Meier curves according to ADT, RT, and RP use for the cumulative probabilities of remaining free of hematologic disorders in general(a); remaining anemia-free (b); and remaining hematologic malignancy-free (c) in the propensity score–matched cohort. ADT = androgen deprivation therapy; RT = radiotherapy; RP = radical prostatectomy.

The Kaplan–Meier disease-free curves for hematologic disorders in the ADT-, RT-, and RP-only groups are shown in Fig 2. The Kaplan–Meier curves showed a lower cumulative probability of remaining hematologic disorders in–free among the patients who were receiving ADT, with the lowest probability observed in those with bone metastasis (*P* < .001).

Analysis of the duration of ADT revealed that the patients who were receiving ADT ≥ 12 months had the highest risk of subsequently developing hematologic disorders (aHR = 1.85; 95% CI = 1.52–2.24; *P* < .001) (Table 3). A significantly increased risk of hematologic disorders was observed with increasing duration of ADT (P for trend < 0.001). According to a further analysis based on the type of ADT, the use of GnRH agonists had a high risk of hematological disorders (aHR = 2.75; 95% CI = 2.13–3.53), followed by oral antiandrogens (aHR = 1.79; 95% CI = 1.45–2.22). Meanwhile, the use of estrogen did not have a significant risk (aHR = 0.82; 95% CI = 0.20–3.31) (Table 4).

**Table 3:** Propensity score-matched Cox regression analysis for the association between the duration of ADT use and hematologic disorders.

	ADT users				*P* for trend
Duration of ADT Use (months)	Crude HR (95% CI)	*P*-value	Adjusted HR (95% CI)	*P*-value	
RP	1 (Ref.)		1 (Ref.)		<0.001**
ADT users					
<12 months ADT use	1.53 (1.22–1.92)	<0.001**	1.56 (1.24–1.97)	<0.001**	
≧12 months ADT use	1.90 (1.60–2.26)	<0.001**	1.85 (1.52–2.24)	<0.001**	

**P <0.001

Abbreviations: RP, radical prostatectomy; ADT, androgen deprivation therapy; HR, hazard ratio; CI, confidence interval

**Table 4:** ADT by type and the risk of subsequent hematologic disorders.

	Hematologic disorders		Anemia		Hematologic malignancy	
ADT exposure	Adjusted HR (95% CI)	*P*-value	Adjusted HR (95% CI)	*P*-value	Adjusted HR (95% CI)	*P*-value
RP	1.00 (ref. group)	--	1.00 (ref. group)	--	1.00 (ref. group)	--
GnRH agonists	2.75 (2.13–3.53)	<0.001**	2.82 (2.18–3.65)	<0.001**	1.10 (0.35–3.46)	0.866
Oral antiandrogens only	1.79 (1.45–2.22)	<0.001**	1.80 (1.45–2.24)	<0.001**	1.76 (0.86–3.60)	0.124
Estrogens only	0.82 (0.20–3.31)	0.774	0.86 (0.21–3.50)	0.836	NA	NA
Other combinations	1.24 (0.96–1.61)	0.104	1.25 (0.96–1.64)	0.101	1.30 (0.52–3.26)	0.578

Abbreviations: ADT, androgen deprivation therapy; RP, radical prostatectomy; GnRH, gonadotropin-releasing hormone; HR, hazard ratio; CI, confidence interval; NA, not applicable

*P <0.05

**P <0.001

## Discussion

To the best of our knowledge, this study first investigated the relationship between the use of ADT and the risk of subsequently developing hematologic disorders, including anemia and hematologic malignancies. This large cohort study enrolled 13,318 PCa patients with a median follow-up period of 4.32 years. A propensity score-matched analysis indicated that patients who received ADT were significantly at increased risk of hematologic disorders compared with those treated with RP.

Several possible mechanisms can explain the association between androgen and hematologic malignancies. First, some studies have shown that androgens inhibit the proliferation of leukemic cell lines in vitro. [18,19] Second, androgen may have effects on telomerase. Anomalies in the TERC/TERT partners of telomerase have been found in hematopoietic myeloid disorders. [18] In relation to this, an increase in telomere length was observed in patients treated with androgen, which induced the hormone-mediated up-regulation of TERT and telomerase enzymatic activity in both lymphocytes and bone marrow cells. [7,20] The use of ADT, which reduces androgen levels, may block this positive effect. Moreover, androgen receptors are widely expressed in the bone marrow and in cells that play a role in human hematopoietic malignancies, including leukemic cells, NHL cells, and Hodgkin’s lymphoma cells. [21,22] Liu et al. have reported a reduced expression of androgen receptors and abnormal methylation of androgen receptors in leukemia cells. [23]

However, the mechanism underlying the association between ADT and non-Hodgkin’s lymphoma remains unclear. In the study of Saylor et al., patients with PCa who received ADT for 12 months had lower levels of interleukin (IL)-6 and higher levels of IL-1α and stromal cell-derived factor (SDF)-1α than those who did not receive ADT. [24] SDF-1α is a chemoattractant factor for myeloma cells in the bone and it increases myeloma cell proliferation. A high level of IL-6 in the serum is associated with poor overall survival among patients with diffuse large B cell lymphoma. [25] Moreover, IL-6 activates the Jak2/STAT3 and PI3K/Akt pathways in mantle cell lymphoma. [26] ADT may reduce the risk of NHL by decreasing IL-6 levels. Mostaghel et al. have shown that the blockade of androgen receptor in mantle cell lymphoma with antiandrogen enzalutamide suppresses cell proliferation in vitro.[27] Our results indicated a higher occurrence of hematologic malignancy with the use of GnRH agonists and oral antiandrogens. Thus, further studies must be conducted to elucidate the relationship between ADT and NHL.

Since the 1960s, androgens have been found to have a therapeutic effect on bone marrow failure syndromes. [7,28] A randomized clinical trial has shown that the addition of androgens to maintenance therapy significantly improves survival in older patients with acute myeloid leukemia. [29]

In 1948, the presence of anemia in male prisoners after castration was discovered. [30] Moreover, anemia is a well-known adverse effect of ADT for PCa. [31] In this study, the RT group was found to be at increased risk of anemia and hematologic malignancies. Exposure to ionizing radiation is a known risk factor for carcinogenesis. RT may damage the DNA and affect leukemogenesis and myelomagenesis. Moreover, RT was found to be associated with the increased risk of leukemia and lymphoma. [11] Bone metastases involving bone marrow infiltration decrease hematopoiesis, leading to anemia and leukopenia. [32]

The present study had some strengths. That is, it was a large-scale cohort study. The NHI system of Taiwan virtually covers all 23 million residents. Moreover, the NHIRD of Taiwan is one of the few nationwide databases in Asian countries.

However, this study also had several limitations. First, data about prostate-specific antigen levels, clinical stages of PCa, and Gleason scores of the patients were not included in the NHIRD database. Second, laboratory data, including hemoglobin levels, were not available. Therefore, we defined anemia according to the use of relevant ICD-9-CM codes and receipt of further treatments. Anemias caused by tumor load particularly in patients with high bone metastatic status were also difficult to identify. The NHIRD data did not distinguish the use of external beam RT and brachytherapy RT. Finally, although the study was conducted for a long period (17 years) and included a large cohort, a longer lag time period may be required for the complete assessment of the incidence of subsequent hematologic malignancies. Thus, further prospective studies must be conducted to investigate the relationship between the use of ADT and hematologic disorders.

## Conclusions

Patients with PCa who received ADT were at increased risk of hematologic disorders. Thus, further studies are warranted, however, to better understand the relationship between ADT and hematologic disorders.

## Supporting information

S1 Table ICD-9 diagnostic codes of diseases and covariates. (DOCX)

S2 Table Multivariable Cox regression analysis for the association of ADT with negative controls. (DOCX)

S3 Table Independent risk factors of hematologic disorders among ADT, RT, and RP by Cox regression analysis. (DOCX)

S4 Table The association between ADT with bone metastasis and hematologic disorders compared to ADT without bone metastasis, RT, RP analyzed by Cox regression model. (DOCX)
