# Supplementary material for: Androgen deprivation therapy for prostate cancer and the risk of hematologic disorders
Source: PLoS One. 2020 Feb 19;15(2):e0229263. doi: 10.1371/journal.pone.0229263 (PMC7029847; doi:10.1371/journal.pone.0229263)
Supplement: S1 Table — (DOCX) [file pone.0229263.s001.docx]

**S1 Table .** ICD-9 diagnostic codes of diseases and covariates

| Diseases and Covariates | ICD-9-CM codes |  |  |
| --- | --- | --- | --- |
| Hematologic malignancy | 200, 201, 202, 203, 204, 205, 206, 207, 208 |  |  |
| Alcohol abuse | 303 , 305.0 , V11.3 |  |  |
| Tobacco use disorder | 305.1, 491.0, 491.2, 492.8, 496, 523.6, 649.0, 989.84, V15.82 |  |  |
| Obesity | 278.0 |  |  |
| Diabetes mellitus | 250 |  |  |
| Hypertension | 401, 402, 403, 404, 405 |  |  |
| Hyperlipidemia | 272 |  |  |
| Coronary heart disease | 410, 411, 412, 413, 414 |  |  |
| Chronic kidney disease | 585, 586, 588 |  |  |
| Chronic liver disease | 456, 571, 572 |  |  |
| Cerebral vascular accident | 430-438 |  |  |
| Crohn’s disease | 555 |  |  |
| Ulcerative colitis | 556 |  |  |
| Rheumatoid arthritis | 714 |  |  |
| Gastrointestinal bleeding | 530.7, 530.82, 531, 532, 533, 534, 537.83, 537.84,578.0, 562.02, 562.03, 562.12, 562.13, 569.86, 569.3, 569.85, 578.1 |  |  |

Abbreviations: ICD-9, International Classification of Diseases-9th revision
