# Supplementary material for: Androgen deprivation therapy for prostate cancer and the risk of hematologic disorders
Source: PLoS One. 2020 Feb 19;15(2):e0229263. doi: 10.1371/journal.pone.0229263 (PMC7029847; doi:10.1371/journal.pone.0229263)
Supplement: S2 Table — (DOCX) [file pone.0229263.s002.docx]

**S2 Table.** Multivariable Cox regression analysis for the association of ADT with negative controls

|  | Adjusted | |
| --- | --- | --- |
| **Outcome** | HR (95% CI) | *P*-value |
| Appendicitis | 0.78 (0.29 - 2.10) | 0.620 |
| Abdominal aortic aneurysm | 1.70 (0.63 - 4.53) | 0.293 |
| Alcoholic cirrhosis | 3.02 (0.29 - 31.18) | 0.354 |
| Necrotizing fasciitis | 0.19 (0.02 - 1.46) | 0.110 |
| Sialadenitis | 1.05 (0.39 - 2.82) | 0.924 |

NOTE. Adjusted for age, and comorbidity diseases such as diabetes mellitus, hypertension, hyperlipidemia, coronary heart disease, chronic kidney disease, chronic liver disease, and cerebral vascular accident.

ADT , androgen deprivation therapy; HR, hazard ratio; CI , confidence interval
