# Supplementary material for: Androgen deprivation therapy for prostate cancer and the risk of hematologic disorders
Source: PLoS One. 2020 Feb 19;15(2):e0229263. doi: 10.1371/journal.pone.0229263 (PMC7029847; doi:10.1371/journal.pone.0229263)
Supplement: S3 Table — (DOCX) [file pone.0229263.s003.docx]

**S3 Table.** Independent risk factors of hematologic disorders among ADT, RT, and RP by Cox regression analysis

|  | | Crude HR  (95% CI) | *P*-value |  | Adjusted HR  (95% CI) | *P*-value |
| --- | --- | --- | --- | --- | --- | --- |
| **Group** | |  |  |  |  |  |
| ADT | 1.65 (1.36 - 2.01) | **<0.001**** |  | 1.60 (1.29 - 1.97) | **<0.001**** |  |
| RT | 2.10 (1.74 - 2.54) | **<0.001**** |  | 1.98 (1.62 - 2.42) | **<0.001**** |  |
| RP | 1.00 (ref. group) | -- |  | 1.00 (ref. group) | -- |  |
| **Age** | | 1.05 (1.04 - 1.06) | **<0.001**** |  | 1.04 (1.03 - 1.05) | **<0.001**** |
| **Comorbidity** | |  |  |  |  |  |
| Alcohol abuse | 0.46 (0.07 - 3.28) | 0.439 |  | 0.48 (0.07 - 3.41) | 0.461 |  |
| Tobacco use disorder | 1.40 (1.16 - 1.70) | **0.005*** |  | 1.16 (0.95 - 1.41) | 0.144 |  |
| Obesity | 0.75 (0.19 - 3.00) | 0.685 |  | 0.93 (0.23 - 3.73) | 0.912 |  |
| Diabetes mellitus | 1.22 (1.02 - 1.47) | **0.027*** |  | 1.15 (0.95 - 1.38) | 0.156 |  |
| Hypertension | 1.06 (0.92 - 1.22) | 0.415 |  | 0.85 (0.73 - 1.00) | 0.053 |  |
| Hyperlipidemia | 1.04 (0.87 - 1.25) | 0.670 |  | 0.98 (0.81 - 1.20) | 0.858 |  |
| Coronary heart disease | 1.21 (1.02 - 1.43) | **0.027*** |  | 1.01 (0.84 - 1.21) | 0.915 |  |
| Chronic kidney disease | 2.21 (1.69 - 2.89) | **<0.001**** |  | 1.88 (1.43 - 2.47) | **<0.001**** |  |
| Chronic liver disease | 0.91 (0.71 - 1.16) | 0.432 |  | 0.91 (0.71 - 1.18) | 0.483 |  |
| Cerebral vascular accident | 1.35 (1.10 - 1.66) | **0.004*** |  | 1.14 (0.92 - 1.42) | 0.232 |  |
| Crohn’s disease | 1.35 (0.67 - 2.71) | 0.395 |  | 1.11 (0.55 - 2.22) | 0.778 |  |
| Ulcerative colitis | 0.00 (0.00 - 999) | 0.962 |  | 0.00 (0.01 - 999) | 0.962 |  |
| Rheumatoid arthritis | 1.75 (1.05 - 2.91) | **0.032*** |  | 1.60 (0.96 - 2.68) | 0.072 |  |
| GI bleeding | 1.54 (1.12 - 2.12) | **0.008*** |  | 1.24 (0.90 - 1.71) | 0.196 |  |
| **Medication use** | |  |  |  |  |  |
| Antiplatelet | 1.46 (1.24 - 1.72) | **<0.001**** |  | 1.23 (1.03 - 1.46) | **0.023*** |  |
| Anticoagulation | 1.16 (0.90 - 1.49) | 0.259 |  | 1.04 (0.80 - 1.34) | 0.780 |  |
| NSAID | 1.25 (1.09 - 1.44) | **0.002*** |  | 1.07 (0.92 - 1.25) | 0.396 |  |

Abbreviations: ADT ,androgen deprivation therapy; RT, radiotherapy ; RP, radical prostatectomy; HR, hazard ratio; CI, confidence interval; GI, gastrointestinal; NSAID, nonsteroidal anti-inflammatory drug.

**P*<0.05 ***P*<0.001
