# Supplementary material for: Androgen deprivation therapy for prostate cancer and the risk of hematologic disorders
Source: PLoS One. 2020 Feb 19;15(2):e0229263. doi: 10.1371/journal.pone.0229263 (PMC7029847; doi:10.1371/journal.pone.0229263)
Supplement: S4 Table — (DOCX) [file pone.0229263.s004.docx]

**S4 Table.** The association between ADT with bone metastasis and hematologic disorders compared to ADT without bone metastasis, RT, RP analyzed by Cox regression model

|  | | Within ADT group  (n=1,797) | | | Total study subjects  (n=5,391) | |
| --- | --- | --- | --- | --- | --- | --- |
| Outcome | | Adjusted HR (95%CI) | *P*-value |  | Adjusted HR (95%CI) | *P*-value |
| Hematologic disorders | |  |  |  |  |  |
| ADT without bone metastasis | 1.00 (ref. group) | - |  | 1.45 (1.17 - 1.81) | 0.008* |  |
| ADT with bone metastasis | 1.94 (1.44 - 2.62) | <0.001** |  | 2.87 (2.08 - 3.97) | <0.001** |  |
| RT | -- | -- |  | 1.98 (1.62 - 2.42) | <0.001** |  |
| RP | -- | -- |  | 1.00 (ref. group) | -- |  |
| Anemia | |  |  |  |  |  |
| ADT without bone metastasis | 1.00 (ref. group) | - |  | 1.50 (1.20 - 1.87) | 0.004* |  |
| ADT with bone metastasis | 1.84 (1.35 - 2.51) | <0.001** |  | 2.78 (1.99 - 3.88) | <0.001** |  |
| RT | -- | -- |  | 1.92 (1.56 - 2.37) | <0.001** |  |
| RP | -- | -- |  | 1.00 (ref. group) | -- |  |
| Hematologic malignancy | |  |  |  |  |  |
| ADT without metastasis | 1.00 (ref. group) | -- |  | 0.98 (0.45 - 2.14) | 0.952 |  |
| ADT with bone metastasis | 2.18 (0.71 - 6.64) | 0.171 |  | 2.50 (0.79 - 7.87) | 0.117 |  |
| RT | -- | -- |  | 2.48 (1.29 - 4.76) | 0.006* |  |
| RP | -- | -- |  | 1.00 (ref. group) | -- |  |

Abbreviations: ADT ,androgen deprivation therapy; RT, radiotherapy ; RP, radical prostatectomy; HR, hazard ratio; CI, confidence interval

**P* <0.05 ***P* <0.001
